# Predictive value of a novel Asian lung cancer screening nomogram based on artificial intelligence and epidemiological characteristics

**DOI:** 10.1111/1759-7714.14140

**Published:** 2021-10-28

**Authors:** Dahai Liu, Xiao Sun, Ao Liu, Lun Li, Shaoke Li, Jinmiao Li, Xiaojun Liu, Yu Yang, Zhe Wu, Xiaoliang Leng, Yang Wo, Zhangfeng Huang, Wenhao Su, Wenxing Du, Tianxiang Yuan, Wenjie Jiao

**Affiliations:** ^1^ Department of Thoracic Surgery The Affiliated Hospital of Qingdao University Qingdao China; ^2^ Health management center The Affiliated Hospital of Qingdao University Qingdao China; ^3^ Department of Thoracic Surgery Qingdao Municipal Hospital Qingdao China; ^4^ Department of Radiology The Affiliated Hospital of Qingdao University Qingdao China; ^5^ Department of IT Management The Affiliated Hospital of Qingdao University Qingdao China; ^6^ Department of Thoracic Surgery Qingdao Chengyang District People's Hospital Qingdao China; ^7^ Department of Thoracic Surgery Shanghai Chest Hospital Shanghai China

**Keywords:** artificial intelligence, Asians lung cancer screening, convolutional neural networks, epidemiological characteristics, nomogram

## Abstract

**Background:**

To develop and validate a risk prediction nomogram based on a deep learning convolutional neural networks (CNN) model and epidemiological characteristics for lung cancer screening in patients with small pulmonary nodules (SPN).

**Methods:**

This study included three data sets. First, a CNN model was developed and tested on data set 1. Then, a hybrid prediction model was developed on data set 2 by multivariable binary logistic regression analysis. We combined the CNN model score and the selected epidemiological risk factors, and a risk prediction nomogram was presented. An independent multicenter cohort was used for model external validation. The performance of the nomogram was assessed with respect to its calibration and discrimination.

**Results:**

The final hybrid model included the CNN model score and the screened risk factors included age, gender, smoking status and family history of cancer. The nomogram showed good discrimination and calibration with an area under the curve (AUC) of 91.6% (95% CI: 89.4%–93.5%), compare with the CNN model, the improvement was significance. The performance of the nomogram still showed good discrimination and good calibration in the multicenter validation cohort, with an AUC of 88.3% (95% CI: 83.1%–92.3%).

**Conclusions:**

Our study showed that epidemiological characteristics should be considered in lung cancer screening, which can significantly improve the efficiency of the artificial intelligence (AI) model alone. We combined the CNN model score with Asian lung cancer epidemiological characteristics to develop a new nomogram to facilitate and accurately perform individualized lung cancer screening, especially for Asians.

## Graphical abstract

A novel AI model was developed for LDCT detection specifically. Our study showed that epidemiological characteristics should be considered in lung cancer screening, which can significantly improve the efficiency of AI model alone. We combined the CNN model score with Asian lung cancer epidemiological characteristics to develop a new Nomogram to facilitate and accurately perform individualized lung cancer screening, especially for Asians.

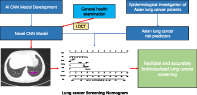

## INTRODUCTION

In 2018, the Global Cancer Statistics Report suggested that there were 18.1 million new cases of cancer and 9.6 million deaths due to cancer globally. Lung cancer has among the highest morbidity (11.6%) and mortality (18.4%) rates, accounting for 1.6 million deaths annually.^1^ Eliminating lung cancer remains a serious challenge.For early‐stage lung cancer, surgery is an effective treatment method: a 75%–100% 5‐year survival rate can be achieved in patients with stage IA non‐small cell lung cancer (NSCLC) after surgery but only a 4% to 17% survival rate for advanced patients.^2^ Therefore, it is crucial to detect and cure the disease in the early stages.

In recent years, evidence from a wide range of sources has indicated that low‐dose computed tomography (LDCT) screening can reduce the mortality of lung cancer.^3^ The National Lung Screening Trial (NLST) revealed a significant 20% reduction in lung cancer mortality with LDCT screening in the USA.^4^ Traditional LDCT screening often produces false‐positive results, 24% of LDCT screening examinations were positive, and the range of false‐positive rates overall was 7.9% to 49.3% for baseline screening.^5^, ^6^ Meanwhile, all of the above assessments require labor‐intensive work from radiologists. Recently, deep learning‐based convolutional neural networks (CNN) have achieved satisfactory effect in image recognition, and several CNN models for chest CT image analysis have been proposed for lung nodule detection and classification.^7^, ^8^ Nonetheless, unlike conventional diagnosis methods, most artificial intelligence (AI) prediction models only consider image features without epidemiological and clinical characteristics. In the conventional process of lung cancer diagnosis, the epidemiological characteristic manifestations of patients are a very important diagnostic basis and must be taken into account. Moreover, there are significant differences between Asians and Europeans and Americans in the epidemiological characteristics of lung cancer.

In this study, we present a prediction model that is derived from a deep learning CNN algorithm on LDCT findings combined with epidemiological characteristics for lung cancer screening, especially for Asians. Finally, a risk prediction nomogram was developed and validated for lung cancer screening in Asian patients with SPN.

## METHODS

### Data sets

We collected an independent data set for CNN model training and testing and named data set 1; the other independent data set named data set 2 and a multicenter data set named data set 3 for the hybrid model training and validation. This study was approved by the institutional review board of The Affiliated Hospital of Qingdao University.

First, we retrospectively collected lung cancer patients' LDCT image data from our institution during January 2014–August 2018 for data set 1. The inclusion criteria of patients included: (1) The patient underwent a general health examination and performed LDCT, pulmonary nodule sizes were less than 30 mm in diameter on LDCT images, (2) histopathological results were confirmed after thoracic surgical resection and the postoperative histopathological result reference standard, and (3) preoperative LDCT could be obtained and the thickness of the LDCT images was ≥5 mm. Ultimately, a total 231 312 LDCT images from 3644 patients were collected for training, tuning, and testing the CNN model.

Second, 790 consecutive patients' preoperative entire volume thoracic LDCT images, clinical data were collected from our institution with the same inclusion criterion as the above between September 2018–December 2019 as data set 2 to train and validate the hybrid model.

In addition, each participant needed to complete an epidemiological questionnaire in a follow‐up.

Third, 210 patients' data were collected by Shanghai Chest Hospital, Xuanwu Hospital Capital Medical University, Qingdao Municipal Hospital, The Affiliated Hospital of Qingdao University and Qingdao Chengyang District People's Hospital with the same inclusion criterion as the training cohort between January 2020 to May 2020 were used for final assessment of the risk prediction nomogram. All patients were screened by a healthy examination and were later diagnosed as requiring surgery by an experienced surgeon in accordance with NCCN guidelines (Vision 1, 2020),^9^ and the pathological results were confirmed after surgery. The details of the three datasets are described and listed in Tables 1, 2, 3.

**TABLE 1:** Data set characteristics of the CNN model training cohort

Male, n (%)	1513, (41.5)
Age (years), mean (SD)	61.042 ± 9.276
Nodule size in diameter (mm), mean (SD)	17.47 ± 6.97
No. of LDCT images	231 312
No. of surgeons	5
Nodule locations, n (%)	
RUL	1153 (31.6)
RML	306 (8.4)
RLL	675 (18.5)
LUL	857 (23.5)
LLL	564 (15.5)
Multilobe	89 (2.4)
Pathological type, n (%)	
Adenocarcinoma	3032 (83.2)
Squamous cell carcinoma	314 (8.6)
Other types	298 (8.2)

Abbreviations: LDCT, low dose computed tomography; LLL, left low lobe; LUL, left upper lobe; No., numbers; RLL, right low lobe; RML, right middle lobe; RUL, right upper lobe; SD, standard deviation.

**TABLE 2:** Data set characteristics of the hybrid model training cohort

(b) Hybrid model training cohort	Malignant nodules (*n* = 501)	Benign nodules (*n* = 255)	*p*‐value
Gender, Male, n (%)	211 (42.1)	128 (50.2)	0.035*
Age, mean, years (SD)	60.66 (9.78)	58.09 (10.49)	<0.001*
Race, Han, n (%)	500 (99.8)	255 (100)	0.475
Marital status, married, n (%)	497 (99.2)	252 (98.8)	0.608
Smoking status, n (%)			<0.001*
No	312 (62.3)	217 (85.1)	
Passive	62 (12.4)	11 (4.3)	
Mild	32 (6.4)	5 (2.0)	
Heavy	95 (16.9)	22 (8.6)	
Alcohol consumption, positive, n (%)	113 (22.6)	67 (26.3)	0.256
Unhealthy dietary habits, n (%)	130 (25.9)	73 (28.6)	0.432
Family history of cancer, n (%)			<0.001*
No	429 (85.5)	250 (98)	
Other cancer history	42 (8.4)	3 (1.2)	
Lung cancer history	29 (5.8)	2 (0.8)	
Dwelling environment exposure, positive, n (%)	16 (3.2)	8 (3.1)	0.967
Occupational exposure, positive, n (%)	35 (7.0)	15 (5.9)	0.564
History of chronic disease, positive, n (%)	243 (48.5)	108 (42.4)	0.109
Pre‐existing lung disease, positive, n (%)	28 (5.6)	10 (3,9)	0.321
Nodule locations, n (%)			0.075
RUL	166 (33.1)	67 (26.3)	
RML	24 (4.8)	21 (8.2)	
RLL	93 (18.6)	63 (24.7)	
LRL	115 (23)	50 (19.6)	
LLL	88 (17.6)	47 (18.4)	
Multilobe	15 (3.0)	7 (2.7)	
Pathological type, n (%)			
Adenocarcinoma	435 (86.8)		
Squamous cell carcinoma	48 (9.6)		
Small cell carcinoma	5 (1.0)		
Large cell carcinoma	1 (0.2)		
Adenosquamous carcinoma	2 (0.4)		
Other types carcinoma	10 (2.0)		
Hamartoma		74 (29.0)	
Papillary adenoma		16 (6.3)	
Inflammation		91 (35.7)	
Sclerosing alveolar tumor		25 (9.8)	
Tuberculosis		14 (5.5)	
Other types benign tumor		35 (13.7)	

*Note*: *p*‐values are derived from the *t*‐test between the malignant and benign groups.

Abbreviations: LDCT, low dose computed tomography; LLL, left low lobe; LUL, left upper lobe; No., numbers; RLL, right low lobe; RML, right middle lobe; RUL, right upper lobe; SD, standard deviation.

* *p*‐value < 0.05.

**TABLE 3:** Data set characteristics of the multicenter validation cohort

Variables	Total	Malignant nodules					Total	Benign nodules					*p*‐value
		XWH	Shanghai	QUAF	QDMH	CYH		XWH	Shanghai	QUAF	QDMH	CYH	
Amount (%)	158 (75.2)	38 (18.1)	39 (18.6)	30 (14.3)	28 (13.3)	23 (11.0)	52 (24.8)	8 (3.8)	17 (8.1)	8 (3.8)	12 (5.7)	7 (3.3)	0.623
Age (SD)	59.6 (11.4)	56 (12.9)	60.3 (10.4)	60.4 (10.2)	59.1 (12.1)	63.04 (10.6)	57.23 (10.8)	55.0 (14.01)	53.8 (10.6)	58.5 (10.53)	61.8 (10.5)	58.1 (7.5)	0.186
Gender, n (%)													0.064
Male	99 (47.1)	20 (43.5)	23 (41.1)	21 (55.3)	19 (47.5)	16 (53.3)	25 (11.9)	2 (4.3)	11 (19.6)	2 (5.3)	7 (17.5)	3 (10.0)	
Female	59 (28.1)	18 (39.1)	16 (28.6)	9 (23.7)	9 (22.5)	7 (23.3)	27 (12.9)	6 (13)	6 (10.7)	6 (15.8)	5 (12.5)	4 (13.3)	
Family history of cancer (%)													0.028*
No	133(63.3)	34 (73.9)	29 (51.8)	26 (68.4)	24 (60)	20 (66.7)	50 (23.3)	8 (17.4)	17 (37.0)	8 (21.1)	11 (27.5)	6 (20.0)	
Other cancer history	13 (6.2)	1 (2.2)	6 (10.7)	2 (5.3)	3 (7.5)	1 (3.3)	2 (1.0)	0	0	0	1 (2.5)	1 (3.3)	
Lung cancer history	12 (5.7)	3 (7.9)	4 (7.1)	2 (5.3)	1 (2.5)	2 (6.7)	0	0	0	0	0	0	
Smoking status, n (%)													<0.01*
No	55 (26.2)	13 (28.3)	16 (28.6)	13 (34.2)	9 (22.5)	4 (13.3)	42 (20)	7 (15.2)	14 (25)	7 (18.4)	8 (20)	6 (20)	
Passive	21 (10.0)	8 (17.4)	5 (8.9)	4 (10.5)	1 (2.5)	3 (10.0)	1 (0.5)	0	1 (1.8)	0	0	0	
Mild	21 (10.0)	2 (4.3)	5 (8.9)	3 (7.9)	5 (12.5)	6 (20.0)	2 (1.0)	0	0	0	2 (5.0)	0	
Heavy	61 (29.0)	15 (32.6)	13 (23.2)	10 (26.3)	13 (32.5)	10 (33.3)	7 (3.3)	1 (2.2)	2 (3.6)	1 (2.6)	2 (5.0)	1 (3.3)	
CNN model score (SD)	86.52 (18.04)	85.3 (17.4)	85.5 (19.4)	88.2 (19.9)	89.7 (14.3)	84.1 (19.04)	64.06 (27.99)	73.9 (16.7)	66.7 (34.0)	64.5 (12.4)	58.4 (31.9)	55.6 (29.7)	<0.01*

*Note*: *p*‐values are derived from the *t*‐test between the malignant and benign groups.

Abbreviations: CYH, Qingdao Chengyang People's Hospital; QDMH, Qingdao Municipal Hospital; QUAF, The Affiliated Hospital of Qingdao University; SD, standard deviation; Shanghai, Shanghai Chest Hospital; XWH, Xuanwu Hospital Capital Medical University.

* *p*‐value < 0.05.

**TABLE 4:** Univariate and multivariate analysis for risk factors of lung cancer screening in training cohort

Variable	Group	Univariate			Multivariate	
		Wald	OR (95%CI)	*p*‐value	OR (95%CI)	*p*‐value
Gender, n (%)	Male	4.448	1.385 (1.023–1.875)	0.035*	2.409 (1.566‐3.705)	0.039*
	Female					
Age, mean, years (SD)	No.	10.747	1.025 (1.010–1.041)	0.001*	1.039 (1.019‐1.059)	0.001*
						
	Other					
Smoking status, n (%)	No	62.8	1 (reference)	<0.001*	1 (reference)	<0.001*
	Passive	12.302	3.758 (1.793–7.874)		4.031 (1.582–10.271)	
	Mild	6.573	3.618 (1.354–9.669)		5.086 (1.150–22.847)	
	Heavy	50.23	6.056 (3.680–9.965)		6.799 (2.907–15.902)	
Alcohol consumption	Positive	1.287	0.817 (0.577–1.158)	0.257	‐	‐
	Negative					
Marital status	Positive	0.26	1.479 (0.329–6.660)	0.61	‐	‐
	Negative					
Family history of cancer	No	20.207	1 (reference)	<0.001*	1 (reference)	0.001*
	other cancer history	11.8	7.946 (2.435–25.925)		8.703 (2.051–36.937)	
	lung cancer history	8.691	8.721 (2.067–32.802)		11.378 (1.685–76.818)	
Dwelling environment exposure	Positive	0.332	1.202 (0.644–2.244)	0.564	‐	‐
	Negative					
Occupational exposure	Positive	0.219	0.795 (0.305–2.077)	0.640	‐	‐
	Negative					
History of chronic disease	Positive	0.102	0.948 (0.682–1.318)	0.75	‐	‐
	Negative					
Pre‐existing lung disease	Positive	0.974	1.450 (0.693–3.035)	0.324	‐	‐
	Negative					
Dietary habits	Positive	0.617	0.874 (0.624–1.224)	0.432	‐	‐
	Negative					
CNN model score	No.	134.359	1.062 (1.051–1.073)	<0.001*	1.084 (1.069‐1.099)	<0.001*

*Note*: *p*‐values are derived from the univariable and multivariable regression analyses among the epidemiological characteristics.

* *p*‐value < 0.05.

### CNN model development

Five experienced radiologists annotated the LDCT images process with LabelImg1.1 software. The labeled lung images were used to train, tune and test the CNN model.

We constructed a 16‐layer feature‐extracted network and a 26 × 26, 52 × 52, 104 × 104 three‐scale detection network based on the framework of the YOLO detection algorithm.^10^ On the premise of extracting enough feature information, the detection speed of the algorithm can be improved by the shallower feature extraction network. The three‐scale detection network can greatly increase the generalization ability of the detection algorithm to the size of the target and improve the recall rate.

**FIGURE 1:**
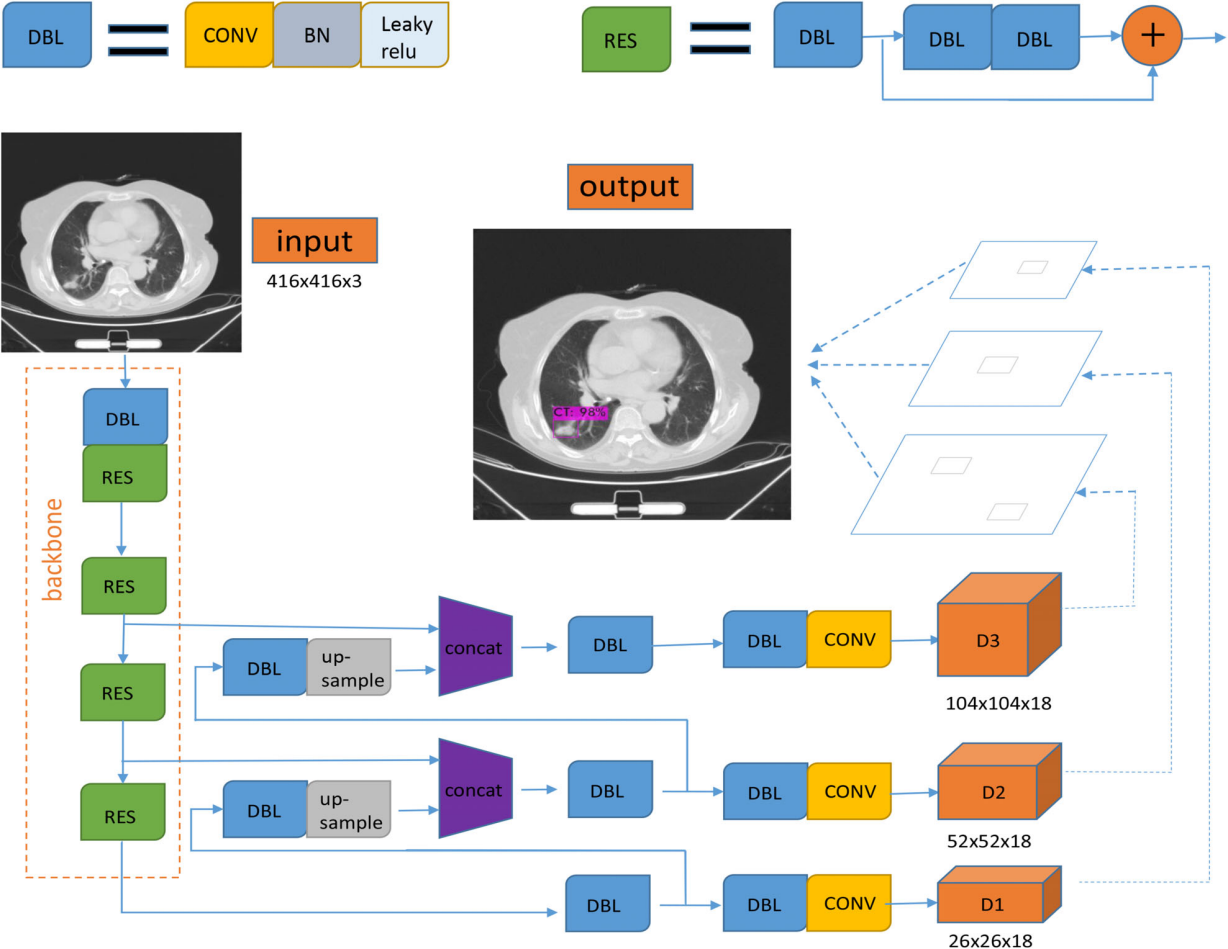
The process of the CNN training. CONV, convolutional layer; BN, batch normalization; leaky ReLU, linear element functions with leak‐correction. The 20‐layer feature extraction network with residual structure was used as the base network. The last three scale feature maps in the basic network were taken as input, and FPN (feature pyramid) structure was used to fuse the features of each layer for detection at three scales. After threshold filtering and nonmaximum suppression (NMS) treatment, the target detection boxes with low confidence were removed and the target detection boxes of the same position and type were fused to obtain the final detection results

The size of the feature graph was 26 × 26, and it was outputted by the feature extraction network with 256 channels. By means of convolution operation, upsampling operation and channel fusion of the feature layer with the same size in the feature extraction network, the detection network further extracted the feature graph. Finally, the feature map of 18 dimensions was formed by three scales. An 18‐dimensional dataset was finally formed by the optimization method of gradient drop with the constraint of the loss function, which contains the data values of three prediction boxes. Each prediction box contains six predictive values, including the confidence of the object in the predictive box, location information of the predicted target point (X, Y), width and height of the target, and classification reliability. Nonmaximum suppression and threshold filtering methods were used to retain the predicted objects with high scores as the detection results (Figure 1).

In the process of forward prediction, each layer of feature map convoluted with convolution kernel pixel by pixel to extract pixel information. Then, batch normalization was used to make the data conform to the normal distribution. The leave relu activation function was used to activate a specific node. The distribution of eigenvalues was fitted by nonlinear function. After the forward prediction, the error between the predicted value and the real value was calculated in the cross entropy loss function. In order to classify the objects better, gradient descent was used to update the parameters of convolution kernel in each layer of neural network for back propagation, and object position information was regressed. The parameters of the trained nonlinear function were saved and the weight file was generated.

### Risk factor screening

In total, epidemiological characteristics were collected from 790 patients. The patients were divided into a malignancy group and benign group according to the postoperative pathology. Currently, postoperative pathology is the reference standard in clinical diagnosis. Epidemiological questionnaires were collected by an experienced surgeon during the follow‐up, including age, gender, race, marital status, smoking status, alcohol consumption, dietary habits, occupational exposure, family history of cancer, nonpulmonary chronic diseases, pre‐existing lung disease, dwelling environment exposure and so on.

Race was divided into Han or others. Marital status was divided into married or unmarried. Smoking status was divided into four categories: no smoking, passive smoking, mild smoking, heavy smoking. According to the definition of WHO, passive smoking meant that non‐smokers had inhaled the smoke exhaled by smokers for at least 15 min more than 1 day in a week. The degree of smoking in current and former smoker was measured by heaviness of smoking index (HSI),^11^ which was <400 for mild smoking, and ≥400 for heavy smoking. Alcohol consumption was assessed using the consumption subscale of the Alcohol Use Disorders Identification Test (AUDIT).^12^ This three‐item subscale assessed participants' frequency and quantity of alcohol use. Each item was scored using a four‐point Likert scale with varying endpoints and summed to create final scores that range from 0 to 12, with higher than eight points defining alcohol consumption positive. Dietary habits were divided into healthy diet or unhealthy diet. A diet of moderation rich in fruits and vegetables was defined as the healthy diet. The occupational exposure positive was defined as work associated with mining and quarrying, metal production industries, including smelting and refining, asbestos production; shipbuilding; and construction. Family history of cancer was divided into three categories: no, other cancer family history and lung cancer family history. Preexisting lung diseases included pneumonia, emphysema, asthma, chronic bronchitis, pulmonary fibrosis, tuberculosis and chronic obstructive pulmonary disease. Dwelling environment exposure positive was defined as residential areas located around heavy industrial factories, mining areas, docks and traffic intensive areas.

### Statistical analysis

Statistical analysis was performed with R software (version 3.6.2; http://www.Rproject.org), MedCalc software and SPSS version 25.0 statistical software (IMB‐SPSS Inc., Armonk, NY, USA). Continuous data were described as means and SD, and categorical variables were described as frequencies and percentages. Continuous variables were compared using the t‐test, and comparisons between two categories were made using Pearson's χ^2^ test. All tests were 2‐tailed and statistical significance was set at *p* < 0.05.

#### Development of a lung cancer risk prediction nomogram

Predictors with *p* < 0.05 screened out by univariate logistic regression analysis were combined with the CNN model score and included in a multivariate logistic regression model. The odds ratios (ORs), probability and 95% confidence intervals (CIs) were estimated for each selected risk factor. The probability score was used to draw the receiver operating characteristic (ROC) curve to assess the sensitivity and specificity of the risk prediction model. Definition of test positivity cutoffs was exploratory. In order to be more convenient for clinical application, we further built a risk prediction nomogram base on multivariable logistic analysis.

#### Performance of the nomogram in the training cohort

The accuracy of prediction model was quantified with the area under the ROC curve (AUC). The statistical significance of the improvement in AUC after adding the risk factors was calculated by Delong's test.^13^ Calibration curves were plotted to assess the calibration of the risk prediction nomogram. Bootstraps with 1000 resamples were applied to these activities.

### Validation of the prediction nomogram

Internal validation. We performed the internal validation using the training data set.

Independent validation. The performance of the internally validated nomogram was examined in the multicenter validation cohort. The logistic regression model trained in the training cohort was applied to all patients in the multicenter validation cohort, and the total score of each patient was calculated. At last, the ROC curve and calibration curve were derived on the basis of the regression analysis.

## RESULTS

### Development of the CNN model

#### Patient characteristics

Details of the CNN model training data and the hybrid model training data are summarized in Table 1. In the CNN model development data set, 58.5% of patients were females and the average age was 61.042 ± 9.276 years. Tumor pathological results revealed that 83.2% of the cases were adenocarcinoma, 8.6% were squamous cell carcinoma, and 8.2% were other types of pulmonary tumors.

#### CNN model training and test

In total, 231 312 entire volume LDCT images were retrospectively collected to train, tune and test the CNN model. Seventy percent of the images were assigned to the training set, and 30% were assigned to the tuning and testing set randomly. When the threshold was set to 0.24, the precision of the tuned CNN model achieved 95%, and the recall achieved 92%. The trained CNN model can achieve a mean average precision (mAP) of 89.95%.

### Development of the lung cancer prediction nomogram

#### Patient characteristics

Patient characteristics in the training cohort are given in Table 2. Finally, a total of 756 patients were retrospectively enrolled and 34 patients were excluded because of LDCT images or follow‐up data absence. There was a total of 63.3% of patients with malignancies with average ages of 60.66 ± 9.78. There were significant differences in age, gender, family history of cancer and smoking status between the malignancy and benign groups (*p* < 0.001). Details of the multicenter validation cohort data are summarized in Table 3. The preoperative LDCT images and epidemiological data of 210 patients were collected from five medical centers. There were 158 malignancy nodules and 52 benign nodules. There was no significant difference in the number of pathological classifications (*p* = 0.623). There were significant differences in the CNN model score, smoking status, and family history of cancer between the two groups (*p* < 0.05).

#### Risk factor screening

Epidemiological characteristics showed an association with postoperative pathological status. By univariate logistic regression analysis, compared with patients with benign pulmonary nodules, patients with malignant pulmonary nodules were more likely to be older, female, tobacco exposure and positive family history of cancer. Furthermore, multivariate logistic analysis identified that age (*p* < 0.001), gender (*p* < 0.001), family history of cancer (*p* = 0.001), smoking status (*p* < 0.001) and CNN model score (*p* < 0.001) were independent risk factors for lung cancer (Table 4).

Development of a prediction model

**FIGURE 2:**
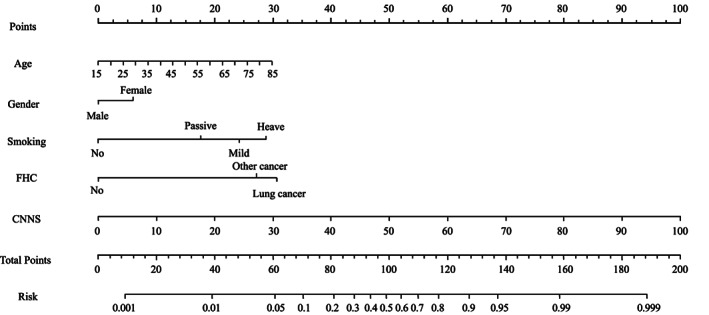
Developed lung cancer prediction nomogram. Smoking, smoking status; FHC, family history of cancer; CNNS, CNN model score. The prediction nomogram was developed in the training cohort, with age, gender, smoking status, family history of cancer and the CNN model score incorporated

The hybrid model that incorporated the independent epidemiological predictors and the CNN model score was established and presented as a nomogram (Figure 2).

### Validation of the prediction nomogram

#### Internal validation

**FIGURE 3:**
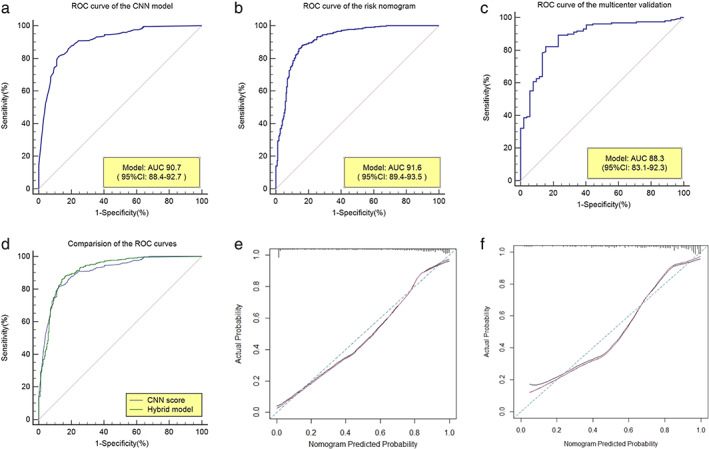
ROC curves and calibration curves of the risk prediction nomogram and the CNN model. (a) ROC curve of the CNN model in the validation cohort. (b) ROC curve of the risk prediction nomogram in the training cohort. (c) ROC curve of the risk prediction nomogram in the validation cohort. (d) Comparison of the ROC curves between the CNN model and the risk prediction nomogram. (e) Calibration curve of the model with addition of epidemiological characteristics in the training cohort. (f) Calibration curve of the model with addition of epidemiological characteristics in the validation cohort. ROC curves showed the AUC of each model in different cohort. The comparison of the AUC was performed between the CNN model and the risk prediction nomogram by Delong's test. Calibration curves showed the calibration of each model in terms of the agreement between the postoperative pathological results and predicted risks of lung cancer. The x‐axis represents the predicted lung cancer risk, the y‐axis represents the actual lung cancer risk. The diagonal blue dotted line represents the consistency between the actual risk and the predicted risk for lung cancer. The amaranth pure solid line reveals the accuracy of prediction of our nomogram, of which a closer fit to the diagonal dotted line indicates that the prediction is more accurate

The training cohort of the 765 patients' data was used as the internal validation data set. The overall predictive accuracy of the nomogram, as measured by ROC curve, was 91.6% (95% CI: 89.4%‐93.5%), indicating good discrimination (Figure 3(b)). The sensitivity and specificity were 86.03% (95% CI: 82.7%‐88.9%) and 85.88% (95% CI: 81.1%‐89.9%), respectively. The calibration plot revealed that the nomogram was well calibrated (Figure 3(e)).

#### Independent validation

Data from 210 patients were used as the independent validation data set from five medical centers. The AUC of the risk nomogram for lung cancer prediction was 88.3% (95% CI: 83.1%‐92.3%) (Figure 3(c)). The sensitivity and specificity were 82.28% (95% CI: 75.4%‐87.9%) and 84.62 (95% CI: 71.9%‐93.1%), respectively (Figure 3). Good calibration of lung cancer prediction probability was observed in a multicenter validation cohort (Figure 3(f)).

### Comparison of the CNN and hybrid models

As shown in Figure 3, the only CNN model had an AUC = 90.7%; with the addition of the epidemiological predictors, the AUC was significantly improved to 91.6% (*p* = 0.00963, Delong's test). This suggests an important role of epidemiological risk factors in the prediction of lung cancer.

## DISCUSSION

We developed and validated a nomogram for lung cancer screening based on the CNN deep learning algorithm and epidemiological characteristics. The nomogram incorporates five items: CNN model score, age, gender, smoking status and family history of cancer. The CNN model successfully classified patients according to their LDCT ‐ image features. Incorporating the CNN model score and epidemiological risk factors into an user‐friendly nomogram assists lung cancer screening. Previous studies showed that the CNN deep learning algorithm could be applied in some diagnosis areas, such as gastric cancer, liver tumor, and skin cancer, and achieved remarkable success.^14^, ^15^, ^16^

In lung cancer, a few researches have previously studied pulmonary nodule detection and classification by CNN. The first report on the application of a deep learning model to nodule classification came from Hua et al.^17^ Encouraging results were revealed in both the deep belief network and CNN models for pulmonary nodule classification. Ardila et al.^18^ suggested a three‐dimensional deep learning CNN model that uses a patient's current and prior CT images to predict the risk of lung cancer. Their model allowed end‐to‐end lung cancer screening and achieved satisfactory result: AUC 94.4%. Nibali et al.^19^ used a deep residual CNN model to detect pulmonary nodules and achieved satisfactory performance (sensitivity: 91.7%, specificity: 88.6%). Most of the above models were mainly applied to the classification of high‐resolution CT images with a thickness of 0.625–1 mm. However, low‐dose spiral CT is commonly used with 5 mm layer thickness images in lung cancer screening. In this study, we specially established a CNN detection model for LDCT images with a thickness of 5 mm.

Compared with those previous deep learning models used to pulmonary nodule detection, our CNN model was trained and verified by the YOLO detection network. YOLO addresses target detection as a regression problem. The YOLO network borrows from the classified network structure of GoogLeNet. The difference is that YOLO does not use the inception module but instead uses a simple replacement of the 1 x 1 convolutional layer plus a 3x3 convolutional layer. YOLO can recognize all the information of the whole image in the process of training and reasoning, and the background false detection rate is low. Tests showed that YOLO's false detection rate for background images was less than half that of Fast RCNN. The source code of YOLO is based on the Darknet framework. The third‐party library is less dependent and easily ported to other platforms such as Windows or embedded devices. Based on these advantages, our CNN model is efficiency, low‐cost, suitable for population screening in various regions, and easy to promote.

In our research, the application of the deep learning CNN model was improved for lung cancer screening using amount LDCT images with matched pathologically confirmed annotations. We achieved 90.7% AUC on the CNN model. However, the lack of epidemiological and clinical data has hindered the development of CNN model, rendering it incapable of comprehensive consideration. In the basis of the CNN model, we further constructed a hybrid model combined other risk factors.

The occurrence of lung cancer is multifactorial,^20^, ^21^, ^22^ and screening for lung cancer requires comprehensive consideration. In particular, epidemiological characteristics of Asians are different from Europeans and Americans.^23^, ^24^ In our study, an epidemiological questionnaire were designed for lung cancer risk factors screening according to the Asians epidemiological characteristics, including genetic factors, behavioral factors, environmental factors and so on. Finally, four independent risk factors were identified by univariate and multivariate analysis.

Smoking is one of the main risk factors for lung cancer. There is a direct correlation between the amount of smoking and the risk of lung cancer.^25^, ^26^ We use the smoking index and secondhand smoke exposure as the subcriteria for smoking‐related risk factors, and smoking was also the most important epidemiological predictor screened in our risk model with an ORs of 4.031, 5.086 and 6.799 in three smoking exposure status respectively. Our study shows that age is an independent factor in the development of lung cancer, with an ORs of 1.039. In Cao's study,^27^ lung cancer was deemed a senescent disease in some ways, with an increased risk of DNA damage due to the constant shortening of telomeres during repeated cell replication cycles. On the other hand, air pollution is a significant risk factor for lung cancer.^28^ Exposure to industrial exhaust, car exhaust, kitchen smoke, or decorative formaldehyde increases the risk of lung cancer with age. In our research, female has more likely to suffer from lung cancer. The reason for this result may be due to the number preponderance of Asian female adenocarcinoma patients in our data. And it has also been proved in studies whether in the Americas or Asia.^29^, ^30^

In our study, we also found that family history of cancer was associated with an increased incidence of lung cancer. Various studies have indicated that patients who have a positive family history of cancer have a significantly higher risk of developing the disease. Matakidou et al.^31^ showed that smokers with a family history of cancer had a two‐fold increased risk of lung cancer. A positive family history of lung cancer showed a 1.5‐fold increase in lung cancer risk among nonsmoking families.^32^ We found that the ORs of a family history of other cancers and lung cancer were 8.703 and 11.378, respectively. This suggests that genetic factors play an important role in the development of lung cancer.

In addition to these factors, dietary habits,^33^ occupational exposure,^34^ pre‐existing lung disease^35^ and so on have also been reported to be associated with the occurrence of lung cancer, but no significant correlation was found in our multivariate analysis. The explanations for the other differences remain to be expounded and may provide new insights into the cause of lung cancer involved. Based on the above previous findings, we attempted to build a prediction model that incorporated the CNN model score and epidemiological characteristics.

Nomograms are graphical tools that use algorithms or mathematical formulae to estimate the probability of an outcome and optimize the prediction accuracy for each patient.^36^ To better use our research in clinical, we further constructed a nomogram that incorporated the four risk factors of CNN model score, age, gender, smoking and family history of cancer, and it showed accuracy and discrimination in predicting the risk of lung cancer, with an AUC of 91.6%. Through this model, clinicians could more precisely assess the risk of lung cancer in the screening population and formulate more precise management measures. For example, consider a male heavy smoker with LDCT screening who is 40 years old, with a positive family history of other cancer and the CNN model score was 80 points. Our nomogram calculations are as follows: age = 40, which corresponds to 11 points; smoking status = high risk, which corresponds to 29 points; family history of cancer = positive family history of other cancers, which corresponds to 27 points; CNN model score = 80%, which corresponds to 80 points; this equals 147 total points, corresponding to a lung cancer probability of 96%. The Youden index of the model was 71.91, and this patient got a positive result. To our knowledge, this is the first nomogram to combine the AI LDCT detection and epidemiological risk factors for lung cancer screening. Our nomogram can conveniently and accurately screening for lung cancer with improved efficiency, low cost, simple procedure, and high scalability.

Nevertheless, there were several limitations in our findings. First, several potential biases were inevitable due to the retrospective design of the study. Second, in the screening of epidemiological characteristics, it was still difficult to fully explain that the model has already contained all the necessary epidemiological characteristics. Some potential risk factors for lung cancer might not be included in our study, such as other environmental pollutants,^37^, ^38^ and the level of education^39^ could not be examined for confounding effects. Finally, as far as epidemiological studies are concerned, the sample size of this study is relatively small. Therefore, we should further increase the sample size and combine more lung cancer risk factors to improve the performance of the model.

In conclusion, our study showed that epidemiological characteristics must be considered in Asians lung cancer screening, which can significantly improve the efficiency of AI model alone image recognition for lung cancer screening. We combined the CNN model score with the epidemiological characteristics to construct a new Nomogram to facilitate and accurately perform individualized lung cancer screening, especially for Asians.

## CONFLICT OF INTEREST

All authors have completed the ICMJE uniform disclosure form. The authors have no conflicts of interest to declare.
